# An adaptive spatiotemporal correlation filtering visual tracking method

**DOI:** 10.1371/journal.pone.0279240

**Published:** 2023-01-06

**Authors:** Yuhan Liu, He Yan, Wei Zhang, Mengxue Li, Lingkun Liu

**Affiliations:** School of Artificial Intelligence, Chongqing University of Technology, Chongqing, China; Mae Fah Luang University, THAILAND

## Abstract

Discriminative correlation filter (DCF) tracking algorithms are commonly used for visual tracking. However, we observed that different spatio-temporal targets exhibit varied visual appearances, and most DCF-based trackers neglect to exploit this spatio-temporal information during the tracking process. To address the above-mentioned issues, we propose a three-way adaptive spatio-temporal correlation filtering tracker, named ASCF, that makes fuller use of the spatio-temporal information during tracking. To be specific, we extract rich local and global visual features based on the Conformer network, establish three correlation filters at different spatio-temporal locations during the tracking process, and the three correlation filters independently track the target. Then, to adaptively select the correlation filter to achieve target tracking, we employ the average peak-to-correlation energy (APCE) and the peak-to-sidelobe ratio (PSR) to measure the reliability of the tracking results. In addition, we propose an adaptive model update strategy that adjusts the update frequency of the three correlation filters in different ways to avoid model drift due to the introduction of similar objects or background noise. Extensive experimental results on five benchmarks demonstrate that our algorithm achieves excellent performance compared to state-of-the-art trackers.

## 1 Introduction

Visual target tracking technology aims to locate a moving target of interest in a video image and then to capture the object’s real-time position, motion state and trajectory information. At present, the direction of the considerable demand for tracking tasks involves online tracking of general objects without specific restrictions and requirements regarding the category, shape, tracking scene, and tracking target. In an actual tracking scene (intelligent traffic monitoring [1, 2], unmanned aerial vehicle (UAV) [3], search-and-rescue missions [4], etc.), the tracking target may experience appearance disturbances from the target background or the target itself, such as sudden changes in illumination, target deformation, similar colors between the target and the background, and target occlusion [5–7]. In addition, most trackers do not make reasonable use of spatio-temporal information during the tracking process. In general, strategies for utilizing limited spatio-temporal information to construct a reasonable target tracking model while avoiding appearance interference during the tracking process is still an unsolved problem.

The discriminative correlation filter-(DCF) based method has attracted extensive attention because of its high accuracy and robustness during tracking. Due to the rapid development of deep learning, deep learning networks are being used to mine high-level semantic features in images to provide better target features for trackers. Most of the current correlation filter trackers use pre-trained convolutional neural networks (CNNs) to extract the targets’ deep features, and after using deep features instead of traditional hand-crafted features, the trackers’ performance significantly improved [8–13]. Another algorithm, the HCF [8] algorithm, uses the deep features of different layers to separately train correlation filters and perform coarse-to-fine fusion. However, HCT does not consider the temporal context during tracking. The MCCT [14] tracker extracts various types of features, trains different correlation filter models, and adaptively selects the model. However, it only uses the adjacent historical frame information to train the correlation filtering model, ignoring the long-distance context information. C-COT [9] and ECO [10] have also achieved very good performance during the same period; these two algorithms use continuous interpolation and filter for joint optimization, but the tracking accuracy cannot be improved by using better deep features [15]. ATOM [16] uses the hard negative mining strategy [17] to update the template while adjusting the learning rate so that the tracking model can quickly adapt to the influence of interference, but the algorithm ignores the impact of occlusion on the training sets. It is therefore easy to mistakenly identify the occluded objects as the tracking target. DiMP [18] removes the hard negative mining strategy, uses a fast update strategy, and performs two recursive optimizations every 20 frames to refine the target model. However, a fixed model update frequency may introduce a large number of meaningless negative samples, which reduces the generalization ability of the model and severely affects the discriminative power of the classifier. Furthermore, contaminated positive samples may cause model degradation and lead to tracking drift. DiMP simply uses the historical frame information to build the tracking model and does not make use of long-distance spatio-temporal information during the tracking process, which may cause the model to lack the ability to effectively handle global context information in complex scenes. Finally, it may lose tracked targets due to tracking challenges such as large deformation of the target body, occlusion by similar objects, and disappearance from the field of view [19].

Aiming at the above problems, we propose a new adaptive target tracking algorithm based on the spatio-temporal correlation filter. Its main idea is to use the Conformer network [20], which performs better in the transformer network [21–23], to extract the tracking target features. Then, to model tracking targets from a different time and space, the initial tracking target is used as the initial template, the current frame tracking search area is used as the search template, and the dynamic target during the tracking process is used as the dynamic template. The three templates train the corresponding correlation filter tracking model to realize three-way tracking; to reasonably select the corresponding model for tracking in different environments, we combine the average peak-to-correlation energy (APCE) [24], peak-to-sidelobe ratio (PSR) [25], and trajectory smoothness degree as the tracking confidence evaluation index and select the tracking result with the best evaluation. At the same time, to quickly adapt to the interference, the tracking state is judged by the tracking confidence proposed in this paper, which adaptively judges whether the model needs to be updated and changes the model’s learning rate. In addition, the Conformer network’s image classification ability is used to control the dynamic template update.

The main contributions of this paper are summarized as follows:

- We propose a dynamic template update method that can flexibly obtain the target’s spatial information and compensate for the defect that some correlation filter tracker only uses historical frame information to make predictions.
- To improve the model’s distractor discrimination, we propose an adaptive model update strategy that further exploits the valuable samples selected by the PSR, APCE, and trajectory smoothness degree.
- In contrast to most of the existing DCF-based tracking methods, which use only single-way correlation filters to achieve tracking (i.e., a single tracking result), we propose a three-way tracking algorithm that enables the tracker to flexibly address appearance changes and geometric deformations of the tracked target over time.

To demonstrate the effectiveness of the tracking framework proposed in this paper, we conduct extensive experiments on the following object tracking evaluation datasets: VOT2020 [26], GOT-10K [27], OTB2015 [28], OTB2013 [29] and LBT50 [30]. The experimental results show that our proposed (ASCF) tracker exhibits excellent performance on four benchmarks.

## 2 Related works

In this section, we review related work on template updating and spatio-temporal information in trackers, and briefly review recent DCFs-based trackers.

### 2.1 Correlation filter tracking

All correlation filter trackers are online training tracking models. As the first CF-based tracker, MOSSE [25] has high accuracy and achieves the fastest tracking speed. HDT [31] introduced deep learning into correlation filtering, and an algorithm that adaptively changes the weight of the filter under each scale feature was designed. [9] and ECO [10], which are representative correlation filter trackers, achieved very good performance over the same period, but they did not achieve further performance gains when using deeper networks. In [15], scholars investigated how to use deeper networks to improve the accuracy and robustness of correlation filter trackers, and to solve the problem of target scale transformation during the tracking process, scholars proposed CFML [32]. In [33], a background-aware correlation filter model with saliency regularization is established to address boundary effects in correlation filter tracking, and another model, CFNet [11], was proposed to embed correlation filtering into a two-way network for end-to-end training and learning. The research team who proposed ECO [10] drew on the advantages of end-to-end trackers such as CFNet and proposed the use of a better gradient descent method to learn a convolution kernel. This strategy is similar to the correlation filter but has the ability to distinguish foreground and background [16, 18], and its performance made it the current state-of-the-art model.

In this work, ECO is selected as our baseline method. Different from the DCF-based trackers mentioned above, we propose a three-way tracker that builds correlation filters in different spatio-temporal areas to obtain multi-tracking results.

### 2.2 Tracking model update

To adapt to the target’s appearance changes, visual tracking algorithms generally adopt the model update strategy. However, there are also trackers [8, 34] that do not use a model update strategy and build a tracking model by using only the initial frame. This type of tracker is prone to tracking drift when the target in the search area undergoes large deformation. ECO [10] proposes a sparse model update strategy and sets a fixed update interval, while LMCF [24] proposes the APCE metric to determine the tracking accuracy and thus uses it to decide whether to update the model. However, APCE may introduce negative samples into the model due to inaccurate judgments about the updates, and many trackers [9, 11, 14, 35–38] that use deep learning networks to extract features ignore the image classification capabilities of the pretrained networks they use.

In contrast, we propose an adaptive model update strategy that can better solve the drift phenomenon during tracking by judging whether the model needs to be updated by combining the pre-training training confidence and feature extraction networks.

### 2.3 Spatio-temporal information in visual tracking

The global context information in the target tracking task includes both temporal information and spatial information. Temporal information involves tracking object state changes across frames; spatial information involves object appearance information and nearby background information. The recently popular offline Siamese trackers [34, 36, 39–41] use only spatial information for tracking and achieve target tracking by using the initial template and the current search area for module matching. In [42], scholars proposed a novel spatial-channel selection and temporal regularized correlation filter (SCSTCF) model that adds spatial-channel constraints to select features along the spatial and channel dimensions, and some trackers [33, 43] enhance the spatio-temporal contextual connections by introducing spatio-temporal saliency. BSTCF [44] introduces background constraints and spatio-temporal regularization to solve the problem that the object background of the traditional CF model is not modeled over time. The CACF [45] tracker, which combines temporal information and spatial information, adds the background near the target to the filter’s learning and introduces spatial templates to the correlation filtering, which better solves the boundary effect [46]. Trackers that combine spatio-temporal information additionally utilize temporal or spatial information to improve the gain, and later works [47–50] have achieved higher robustness.

Although trackers have made some progress in terms of making full use of spatio-temporal information in recent years, most trackers use convolutional features, which have a limited receptive field and lack the ability to model long-distance spatial relationships. We use the Conformer network instead of convolutional neural networks to extract tracking object features, use the self-attention mechanism to capture long-distance spatial relationships [23], and capture the tracking target’s appearance changes and background changes through dynamic templates to increase the utilization of spatial information.

## 3 Proposed visual tracking methods

**Fig 1:**
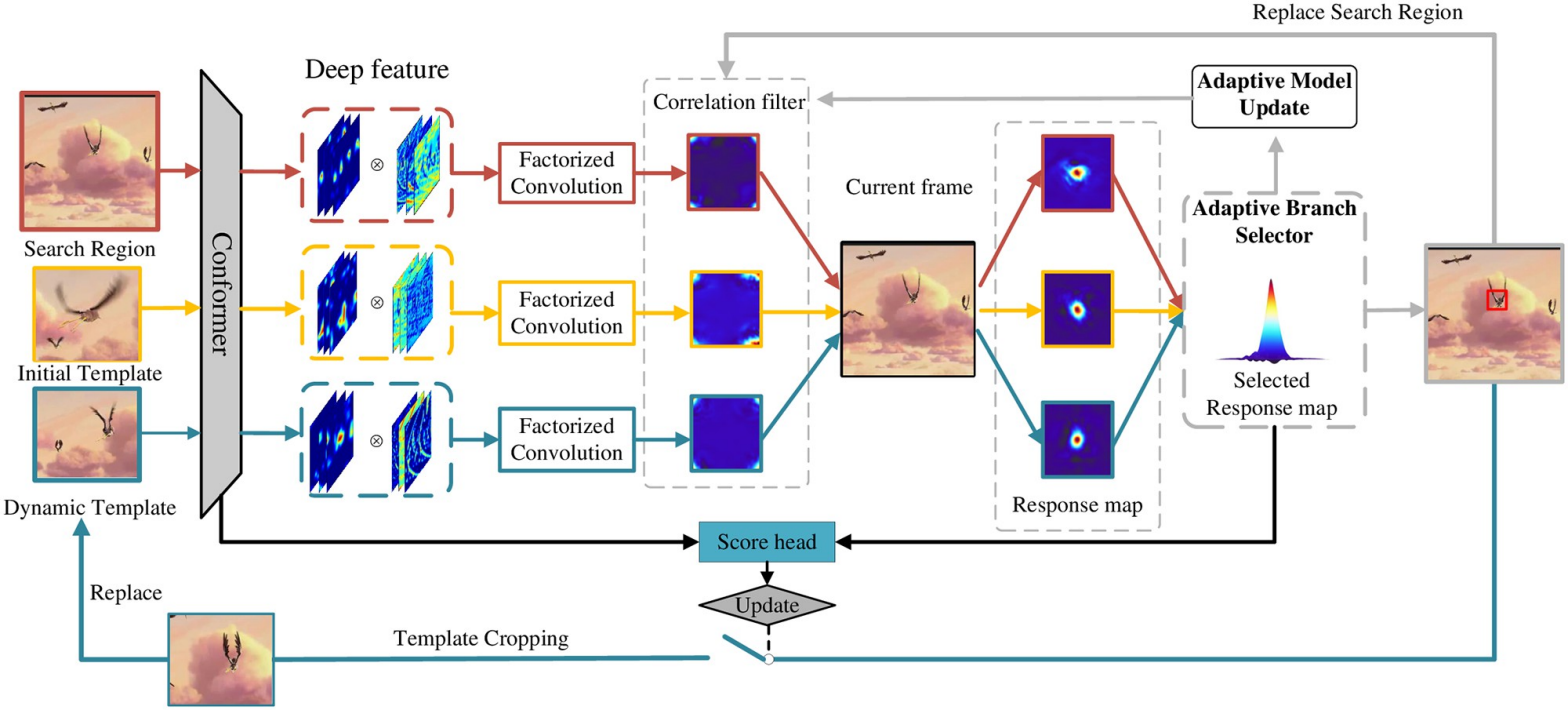
Pipeline of the proposed tracking framework. The initial template refers to initial frame target. The dynamic template is sampled from intermediate frames.

In this section, we describe the proposed ASCF algorithm in detail. ASCF adopts the Conformer backbone network for feature extraction and constructs three-way parallel correlation filters for tracking, as shown in Fig 1. Unlike most deep correlation filter trackers, ASCF can extract global and local features due to our choice of backbone network; at the same time, ASCF uses the Conformer [20] network’s classification ability to judge the state of dynamic templates and correlation filter models for updating. Three different correlation filters are trained by different spatio-temporal target features, and then, the best correlation filter model is selected for tracking through joint evaluation by the APCE and PSR. Different tracking models are reasonably selected for different tracking object states to improve the tracking accuracy.

Through the above design, our tracker can reasonably select the correlation filtering model for tracking according to the tracking scene and update the model at the appropriate time to reduce the error caused by invalid model updates.

### 3.1 Conformer backbone

The quality of the captured features is very important because the target can change greatly over time during tracking. The self-attention mechanism in the Conformer network can capture the global features that are ignored by the convolutional neural network and improve the feature quality. Consistent with correlation filter trackers based on deep learning [8, 31], our proposed ASCF accepts the surrounding images of the tracked object as the input to the backbone network. Overall, there are three inputs to the backbone network: the template image of the initial target object $z\in R^{3\times H_{z}\times W_{z}}$, the search image for the current frame $x\in R^{3\times H_{x}\times W_{x}}$, and the dynamically updated dynamic template image $d\in R^{3\times H_{d}\times W_{d}}$. During the tracking phase, only the search images and dynamic template images are input.

Conformer adopts a concurrent structure and builds CNN and Transformer branches. First, a 7×7 convolution with a stride of two followed by a 3×3 max pooling layer, which also has a stride of two, is used to extract shallow feature maps *w*_*stem*_. hese shallow features are rich in texture and contour information.

First, the image needs to be convoluted and normalized as shown in Eq (1), which is expressed as follows:

$$\begin{array}{c} y=\frac{\text{conv}(x)-\text{mean}(\text{conv}(x))}{\sqrt{\text{Var}(\text{conv}(x))}+\zeta } \end{array}$$ (1)

where *x* represents the input image (search template), conv represents the convolution operation, and mean and Var denote the mean and variance of the calculation. *ζ* increases the value to *e*^−5^ to avoid the denominator being zero.

After normalization, a 64-channel feature map is obtained using the ReLU activation function and max pooling as follows:

$$\begin{array}{c} w_{\text{stem}\;}=\text{Max}\;\text{Pool}\left(\text{ReLU}\left(y\right)\right) \end{array}$$ (2)

To obtain local and global features at the same time, a feature coupling unit (FCU) is used to continuously couple local features and global representations in an interactive manner. When the feature map extracted by the CNN enters the Transformer branch, a 1×1 convolution is used to make the feature map consistent with the number of patch embedding channels (the number of channels is 384). Then, a 4×4 downsample with a stride of four is used, and we also add a (1, 384) dimension class_token to complete the spatial alignment. The shape of the patch is *nE*, and the fusion process is shown in Eq (3), which defines patch i in *P*_*c*_ (denoted as $P_{c}^{i}$) and patch j in *P*_*t*_ (denoted as $P_{t}^{i}$) as follows:

$$\begin{array}{c} P_{t}^{j}=P_{t}^{j}+\text{Softmax}(\frac{(P_{t}^{j}W_{q}){\left(P_{c}^{i}W_{k}\right)}^{T}}{\sqrt{E}})(P_{c}^{i}W_{v}) \end{array}$$ (3)

where *K* and *E* represent the number of patch embeddings (called *P*_*t*_) and the channel dimension of the transformer branch, respectively. The feature map is divided into *K* patches of 14×14, denoted by $P_{c},W_{q},W_{k},W_{v}\in R^{3\times H\times W}$,, which are learned linear transformations that map the input and $P_{t}^{j}$ to query Q, key K and value V.

When transitioning from the Transformer channel back to the CNN channel, upsampling is performed, and the same attention weights that were employed by the Transformer channel are used, as denoted in Eq (4), which is expressed as follows:

$$\begin{array}{c} {\tilde{P}}_{c}^{i}={\tilde{P}}_{c}^{i}+\text{Softmax}{\left(\frac{(P_{t}^{j}W_{q}){\left(P_{c}^{i}W_{k}\right)}^{T}}{\sqrt{E}}\right)}^{T}{\tilde{P}}_{t}^{j} \end{array}$$ (4)

where ${\tilde{P}}_{c}^{i}$ belongs to $P_{c}^{i}$ and is processed by the convolutional layer, and ${\tilde{P}}_{c}^{j}$ belongs to $P_{t}^{j}$ and is processed by the Transformer block. The feature map *w*_*conv*_*trans*_10_ which is rich in global and local features, can be obtained through the convolution operation.

Most DCF-based trackers [8, 10, 16] do not use the backbone network’s classification function after extracting the target features because these trackers ignore the pre-trained backbone network’s classification ability. Conversely, after the image is entered into the network, we use the pre-trained network’s classification function to provide judgments for both subsequent dynamic template updates and model updates. The proposed model performs global pooling on the CNN branch, obtains the class token [51] of the Transformer branch, and then calculates the classification prediction score using a top-k list as follows:

$$\begin{array}{c} S_{c}=\text{Top}(LN(z_{t}))+\text{Top}(LN(z_{c})) \end{array}$$ (5)

where *z*_*t*_ represents the Transformer branch’s classification, *z*_*c*_ represents the CNN branch’s classification, and LN refers to layer normalization. Finally, the predicted score for image classification is obtained.

### 3.2 Three-way parallel correlation filter tracking

**Fig 2:**
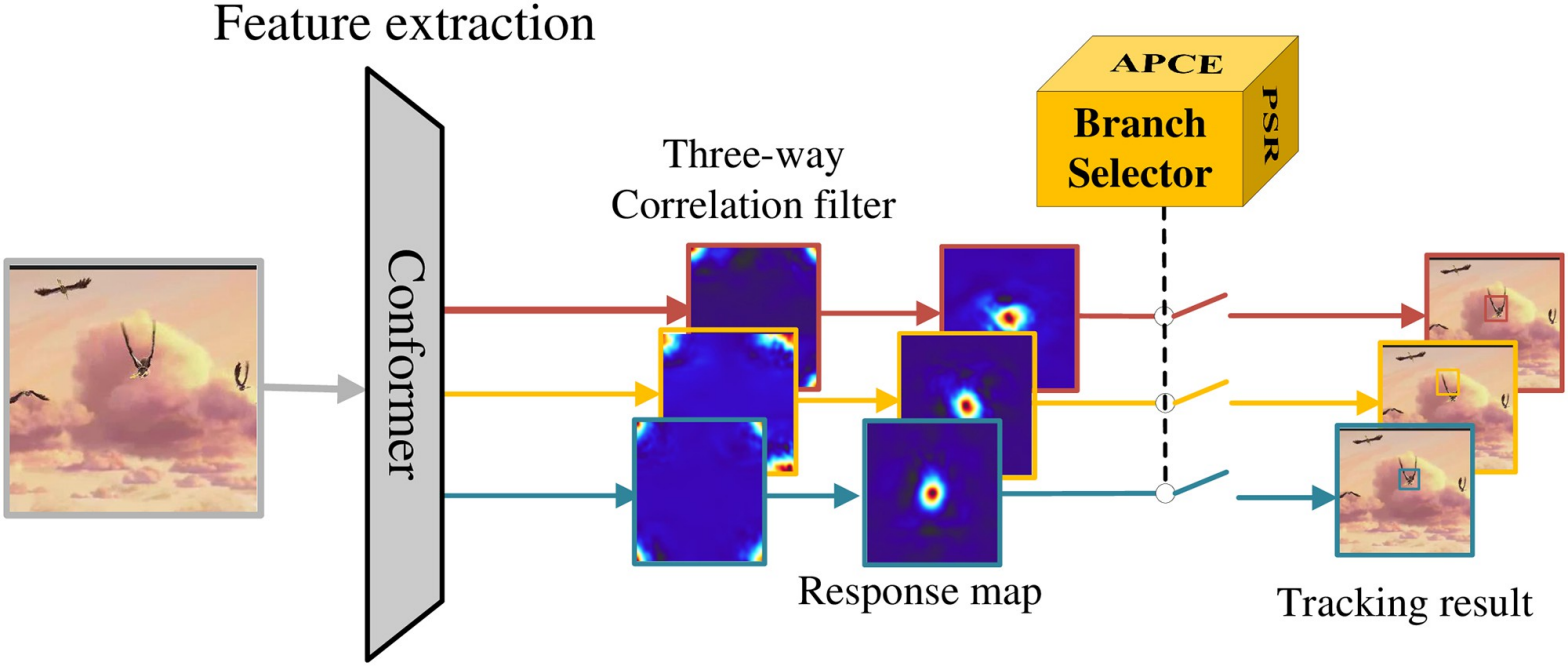
Three-way parallel correlation filter tracking.

In this subsection, we describe how to implement tracking by using three-way parallel correlation filtering. Conventional correlation filter trackers use a single correlation filter to achieve tracking [25], but if they fail to track at a certain frame, it will cause subsequent continuous tracking failures until the tracking target is lost. To improve the tracker’s performance under disturbed conditions such as deformation or occlusion of the tracking target, ASCF uses initial templates (initial frame templates), dynamically updated templates, and search templates to train different correlation filtering models. Subsequent tracking is conducted through the branch selector module, which adaptively selects the model, as shown in Fig 2.

#### 3.2.1 Training correlation filter tracker

All three-way filters are trained in the same way. In this section, using the search template as an example, we explain how to obtain the response map. Suppose that while tracking the t-th frame, the current frame search template is used to train the correlation filter. The search region of the current frame image is considered the detection region $x_{t}\in R^{3\times H_{x}\times W_{x}}$ of the tracking target. The image is then input into the Conformer network, and the features *x*_*j*_ of the *Conv*_*stem* and *Conv*_*trans*_10 layers are extracted as the training samples for correlation filtering. Assuming that there are m training samples in total, the continuous convolution operator [9] is used to transform the features into the continuous space domain. Sample *x*_*j*_ contains a total of D feature channels, and the resolution of sample $x_{j}^{d}$ of the d-th feature channel is *N*_*d*_. $x_{i}^{d}\left[n\right]$ represents a variable in discrete space, and the sample space is expressed as $\chi =R^{N_{1}}\times \ldots \times R^{N_{D}}$.

The interpolation operator $J_{d}:R^{N_{D}}\to L^{2}\left(T\right)$ is used to transform the feature discrete space into the continuous interval [0,T]⊂R, and the *J*_*d*_ (*x*^*d*^) (*t*) interpolation operator is shown in Eq (6), which is expressed as follows:

$$\begin{array}{c} J_{d}(x^{d})\;\left(t\right)=\sum_{n=0}^{N_{d}-1}x^{d}\left[n\right]b_{d}\;(t-\frac{T}{N_{d}}n) \end{array}$$ (6)

The interpolation function *b*_*d*_ is constructed from the standard cubic spline interpolation kernel, as denoted in Eq (7), which is defined as follows:

$$\begin{array}{c} b\left(t\right)=\{\begin{array}{cc} {\left(a+2\right)|t|}^{3}-\left(a+3\right)t^{2}+1 & |t|\leq 1\\ {a|t|}^{3}-5at^{2}+8a\left|t\right|-4a & 1<|t|\leq 2\\ 0 & |t|>2 \end{array} \end{array}$$ (7)

The confidence function *S*_*f*_ uses the convolution filter *f* = (*f*^1^, …, *f*^*D*^) ∈ *L*^2^(*T*)^*D*^ as the parameter, where *f*^*d*^ ∈ *L*^2^(*T*) is the feature filter for channel *d*.

$$\begin{array}{c} S_{f}\left\{x\right\}=\sum_{d=1}^{D}f^{d}*J_{d}\{x^{d}\},x\in \chi \end{array}$$ (8)

The filter *f* minimizes the following function through m pairs of training samples ${\left\{(x_{i},y_{i})\right\}}_{1}^{m}\subset \chi \times L^{2}\left(T\right)$. Eq (9) is minimized by m training samples ${\left\{(x_{i},y_{i})\right\}}_{1}^{m}\subset \chi \times L^{2}\left(T\right)$ to obtain filter *f* as follows:

$$\begin{array}{c} E\left(f\right)=\sum_{j=1}^{m}\alpha_{j}{∥S_{f}\{x_{j}\}-y_{j}∥}^{2}+\sum_{d=1}^{D}{∥\omega f^{d}∥}^{2} \end{array}$$ (9)

Label *y*_*j*_ is the expected output after applying *S*_*f*_ {*x*_*j*_} to the training sample *x*_*j*_, and the calculation of the *ω* penalty coefficient is consistent with [46]. After obtaining filter *f*, the confidence response *S*_*f*_ can be calculated, and we employ the Gauss-Newton method to optimize the function as shown in Eq (9).

#### 3.2.2 Multi-model adaptive selection

When the training of the three-way correlation filters is completed, tracking can be achieved by adaptively selecting the correlation filters. The current frame image is input, the correlation filter response *S*_*fd*_ is calculated and tracked by the dynamic template correlation filter model, the correlation filter response *S*_*fi*_ is tracked by the initial template correlation filter model, and the correlation filter response *S*_*fs*_ is tracked by the search template correlation filter model, which selects an appropriate correlation filter for tracking.

Among the correlation filters, the PSR can represent the peak sharpness of the correlation filter response (CFR), which is used to evaluate the status of the tracked target and the severity of the interference.

$$\begin{array}{c} PSR(S_{f})=\frac{\text{max}\;\{S_{f}\}-\mu \;(S_{f})}{\sigma \;(S_{f})} \end{array}$$ (10)

where max{*S*_*f*_} is the maximum value of *S*_*f*_ in the correlation filter response, and *μ* (*S*_*f*_) and *σ* (*S*_*f*_) are the mean and standard deviation of *S*_*f*_, respectively.

**Fig 3:**
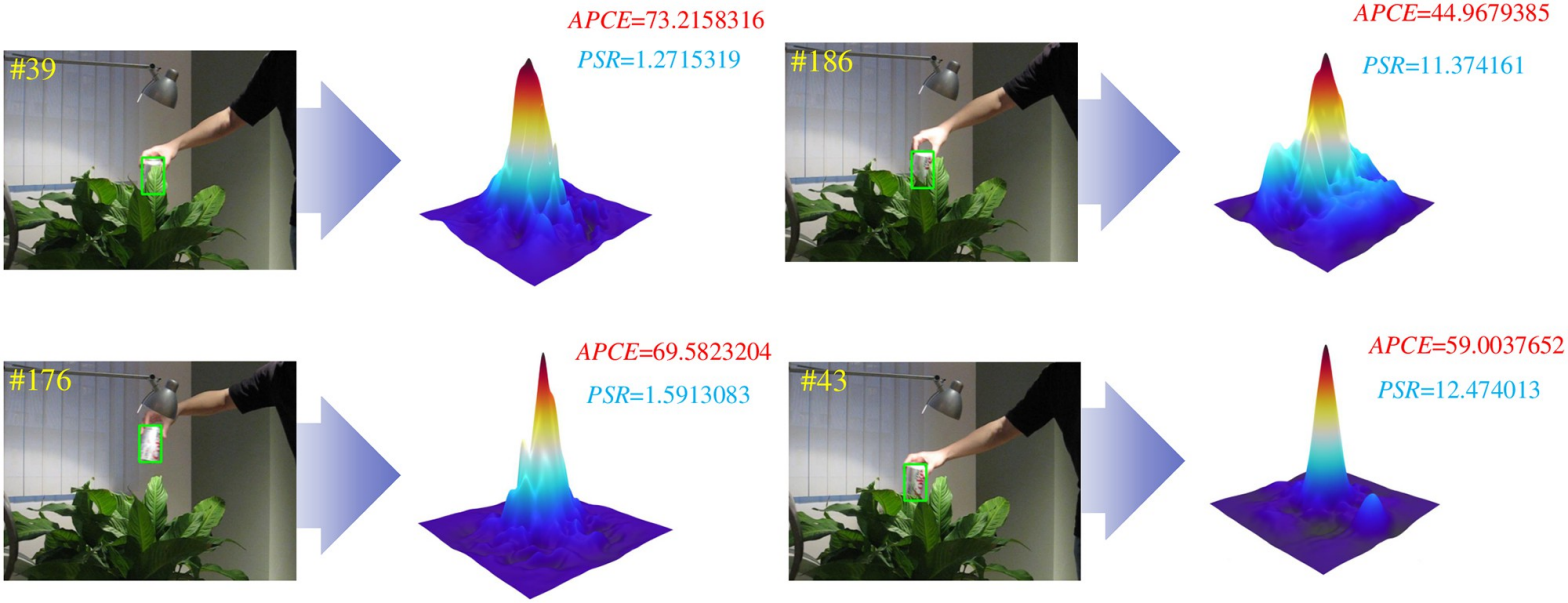
PSR value and APCE value in different tracking environments.

The larger the PSR value, the higher the target tracking confidence and vice versa. However, simply using the PSR to evaluate the target tracking confidence is not sufficient. As shown in Fig 3, at frame 39, PSR==1.2715319. However, at frame 176, the tracking target exhibits dynamic blur and occlusion, among other challenges, and the PSR does not change significantly. But at frame 186 and frame 43, the APCE value is too sensitive.

The APCE [24] evaluation index represents the smoothness of the response graph and is defined in Eq (11) as follows:

$$\begin{array}{c} APCE\;(S_{f})=\frac{\left|\text{max}\;\{S_{f}\}-\text{min}\;\{S_{f}\}\right|}{\text{mean}\;(\sum_{w,h}(S_{f_{w,h}}-\text{min}\;\{S_{f}\}))} \end{array}$$ (11)

where max{*S*_*f*_}, min{*S*_*f*_}, and $S_{f_{w,h}}$ are the maximum response value, minimum response value and corresponding position response value respectively. When the peak is sharper and there are fewer interfering peaks, the APCE will be relatively improved, which will be evident in outcomes such as a smooth response graph with only a single peak. Otherwise, when objects are occluded or missing, multimodal responses appear, and the APCE will decrease significantly.

According to the above analysis and to evaluate the target tracking confidence more reliably, we combine the APCE and PSR as the confidence evaluation indexes of the target tracker to construct a binary function *f*(*PSR*, *APCE*), which is defined as follows:

$$\begin{array}{c} f(PSR,APCE)=\left(1-\gamma \right)\cdot APCE+\gamma \cdot PSR \end{array}$$ (12)

where *γ* ∈ [0, 1] represents the evaluation weights.

The trajectory smoothness degree indicates the reliability of the tracking results to some extent. To make full use of the historical frame prediction information, we measure the target motion trajectory smoothness degree; by measuring the trajectory smoothness degree between the current frame and the previous five frames, the overall trajectory smoothness degree *W* is established as follows:

$$\begin{array}{c} W^{j}=\text{exp}(-{\left(\sum_{i=j-5}^{j}∥\frac{Q^{i}-Q^{i-1}}{\sqrt{2}\theta_{i}\times \eta^{i}}∥\right)}^{2}) \end{array}$$ (13)

where *j* is the current frame number, and *Q*^*i*^ is the center position information of the predicted rectangle frame of the ith frame. *θ*_*i*_ is the mean value of the height and width of the frame’s predicted rectangular frame. Due to a possible low correlation between the current frame and the previous frame, we set the correlation coefficient *θ*. *θ*^*i*^ = 2, *θ*^*i*−1^ = 4, *θ*^*i*−2^ = 8, *θ*^*i*−3^ = 16, *θ*^*i*−4^ = 32, *θ*^*i*−5^ = 64. The score of the current branch at the current frame *j* is calculated as follows:

$$\begin{array}{c} F_{j}=\left(1-\lambda \right)\cdot f_{j}(PSR,APCE)+\lambda \cdot W^{j} \end{array}$$ (14)

where *λ* ∈ [0, 1] represents the evaluation weights.

The model then calculates the PSR, APCE, and trajectory smoothness degree of each branch and substitutes these values into Eq (14) to select the tracker with the highest *F*_*j*_.

### 3.3 Adaptive update strategy

During the tracking process, if the target deforms too fast, it will cause motion blur, and the tracking frame will drift because the model update after each matching will be inaccurate. If the model’s learning rate is low, it can only learn a small part of the information in the current frame, and the model will contain more information from previous frames. In the case of a large number of occlusions, if the model is updated, many negative samples will be introduced, and the model will not be able to effectively handle the global information in complex scenes or perform robust positioning of the target object. Therefore, it is necessary to be cautious while updating the model and only update it in the appropriate tracking situation.

#### 3.3.1 Update of dynamic template

We judge whether the dynamic template needs to be updated using the classification prediction score and classification prediction category calculated by Eq (5), and we set a reliability threshold *δ*. If the classification prediction score is greater than the threshold and the classification prediction category is consistent with the category of the last dynamic update template, then it will perform an update; otherwise, it will not be updated. In addition to the spatial information provided, the dynamically updated template can also capture the temporal changes in the target’s appearance over time, thus providing additional temporal information.

$$\begin{array}{c} dy_{t}=\{\begin{array}{c} dy_{\text{n}\;}\;S_{c}>\delta \;\text{and}\;cgy_{\text{n}\;}=\text{c}gy_{\text{o}}\\ dy_{\text{o}}\;\text{otherwise}\; \end{array} \end{array}$$ (15)

where *S*_*c*_ refers to the classification prediction score calculated in Eq (5), *δ* refers to the threshold, *cgy*_n_ refers to the current frame tracking target category, and *cgy*_o_ refers to the last updated dynamic template classification category.

#### 3.3.2 Model update strategy

Selectively updating the target’s tracking model can improve the tracking efficiency and eliminate the influence of negative samples on the model. In this paper, an adaptive update strategy is proposed to refer to the historical average PSR and APCE values of each tracker, and only when the *f*(*PSR*, *APCE*) of the current frame exceeds the historical frame by a certain percentage is the current frame considered a model update sample; the model is then updated. In this way, the time information can be fully utilized, and the model can be updated and judged in combination with the historical frames. The specific learning rate adjustment steps are as follows: First, a fixed base learning rate Lb is set. Then, the ratio of the current frame’s APCE value to the average APCE value of the historical frame is calculated, and this value is multiplied by Lb to obtain the latest learning rate. We set a fixed threshold, and if the APCE ratio is lower than the threshold, the learning rate is adjusted to 0. This process is expressed in Eq (16) as follows:

$$\begin{array}{c} l=\{\begin{array}{c} L_{b},A_{t}\geq 1\\ L_{b}*A_{t},1>A_{t}\geq A_{T}\\ 0,A_{t}<A_{T} \end{array} \end{array}$$ (16)

where *l* is the updated learning rate, and *A*_*t*_ is the ratio of the APCE value at the current moment to the average APCE value of the historical frame.

Furthermore, the three-way correlation filtering model update strategy is as follows: For the initial template branch, the model is not updated during the entire tracking process ${\hat{x}}_{i}={\hat{x}}_{i}$. The ASCF only updates the model for the remaining two-way correlation filtering.

- For the dynamic update template branch, if the dynamic update template is updated and *f*_*t*_ (*PSR*, *APCE*) ≥ *f*_*T*_ (*PSR*, *APCE*), we update the dynamic update model ${\hat{x}}_{d}^{p}=\left(1-l\right){\hat{x}}_{d}^{p-1}+l{\hat{x}}_{d}$.
- For the search template branch, an update judgment is performed once in five frames, and if *f*_*t*_ (*PSR*, *APCE*) ≥ *f*_*T*_ (*PSR*, *APCE*) is satisfied, ${\hat{x}}_{s}^{p}=\left(1-l\right){\hat{x}}_{s}^{p-1}+l{\hat{x}}_{s}$.

The model is updated according to the learning rate obtained by Eq (16), and the newly-learned model is used to track the next image frame. ${\hat{x}}_{i}$ represents the initial template tracking model, ${\hat{x}}_{d}$ represents the dynamically updated template tracking model, ${\hat{x}}_{s}$ represents the search template tracking model, *f*_*t*_(*PSR*, *APCE*) is the frame calculation of t-th, *f*_*T*_(*PSR*, *APCE*) is the historical calculation mean, and ${\hat{x}}^{p-1}$ is the last updated target model.

**Algorithm 1**: ASCF

**Input**: Sequence frames (*t*-th frame, total of *T* frames). Initial bounding box of target.

**Output**: Target bounding box.

**for** *t* = 1 to *T* **do**

 Extract search region feature map *w*_*stem*_, *w*_*conv*_*trans*_10_ by Conformer

 **if** *t* = 1 **then**

  Using feature maps to training initial template correlation filters by Eq (6) and Eq (9)

 **end**

 **if** *t* > 4 **then**

  Using feature maps to training dynamic tracking template correlation filters by Eq (6) and Eq (9);

  Determine whether the dynamic template needs to be updated by Eq (15);

 **end**

 Using feature maps to training search template correlation filters by Eq (6) and Eq (9);

 Get the dynamic tracking template, initial template and search template confidence response map *S*_*fd*_, *S*_*fi*_, *S*_*fs*_ by Eq (8);

 Select the appropriate tracking template for tracking by Eq (14);

 Update training learning rate by Eq (16);

 Update the dynamic, initial, search tracking model.

**end**

**return** Target bounding box.

### 3.4 Tracking pipeline

We provide a brief overview of this paper’s algorithm in Algorithm 1. This algorithm consists of two main modules: adaptive selection of three correlation filters for tracking and an adaptive update strategy. During the tracking process, the target features are extracted through the Conformer network. Then, the correlation filter is trained, and the highest correlation filter from among the initial, dynamic and search branches is selected for tracking evaluation, and the generated tracking frame is used as prior knowledge to crop the current frame as a reference to generate a search tracking template. During the model’s update process, the dynamic template is checked and updated at the same time. Selectively updating the target’s tracking model not only improves the tracking efficiency but also effectively excludes the impact of negative samples on the mode.

## 4 Experiments

This section introduces the implementation details of our proposed algorithm, ASCF, and then, we conduct comparative experiments with the current state-of-the-art trackers on target tracking evaluation datasets to prove the superiority of this algorithm. The datasets are as follows: OTB2013, OTB2015, VOT2020, GOT-10K and LBT50. Finally, through ablation experiments, the effectiveness of each tracker module is analyzed.

Our tracker was implemented based on Python3.7 and Pytorch1.7.1 and was tested on a desktop computer using a single NVIDIA GeForce GTX 3070 GPU with a 3.7GHz AMD Ryzen 5 5600X CPU. The Conv_stem and Conv_trans_10 layers of the Conformer network are extracted as the features of the tracking target, and we set the search region to 4.5 times the tracking box size during detection. The dynamic template is acquired for the first time after four tracking frames, and the dynamic update template is updated after the 4-th frame. The classification confidence threshold *δ* of the dynamic template is 8.5, as shown in Eq (15). Based on [10], we set the learning rate to 0.01, which is *L*_*base*_ in Eq (16). In Eq (16), the learning rate adjustment threshold *A*_*T*_ is 0.65, and in Eq (12), the weight adjustment coefficient *γ* is set to 0.9 because the APCE value is usually much larger than that of the PSR. In addition, the *λ* in Eq (14) is set to 0.6. To provide a fair comparison, all of the following experiments were performed under identical training settings.

### 4.1 Comparison with the SOTA trackers

#### 4.1.1 OTB-2013

OTB-2013 [29] consists of 51 video sequences and is currently one of the most widely tested datasets in the field of visual tracking. This dataset uses the one-pass evaluation (OPE) [29] protocol as the tracking evaluation indicator.

We compare the ASCF trackers with many state-of-the-art trackers, including MCCT [14], Ocean [40], ATOM [16], UDT [52], DaSiamRPN [17], SiamBAN [53], SiamRPN++ [54], ECO [10] and C-COT [9] on OTB2013. We report two metrics, the area-under-the-curve(AUC) score and the distance precision(DP) score.

The center location error (CLF) is the Euclidean distance from the ground-truth center position (*x*_*g*_,*y*_*g*_)to the predicted center position (*x*_*p*_, *y*_*p*_), as shown in Eq (17). The DP is the percentage of the number of frames whose CLF is greater than a certain distance error threshold, known as the location error threshold (LET), to the total number of frames in the video sequence.

$$\begin{array}{c} C_{LF}=\sqrt{{\left(x_{p}-x_{g}\right)}^{2}+{\left(y_{p}-y_{g}\right)}^{2}} \end{array}$$ (17)

The overlap rate accuracy (OP) refers to the percentage of the number of frames where the overlap rate *ϕ* of the tracking target frame *R*^*P*^ and the ground-truth bounding box *R*^*G*^ is greater than the overlap rate threshold (OT) to the total number of frames.

$$\begin{array}{c} \phi =\frac{\left|R^{P}\cap R^{G}\right|}{\left|R^{P}\cup R^{G}\right|} \end{array}$$ (18)

**Fig 4:**
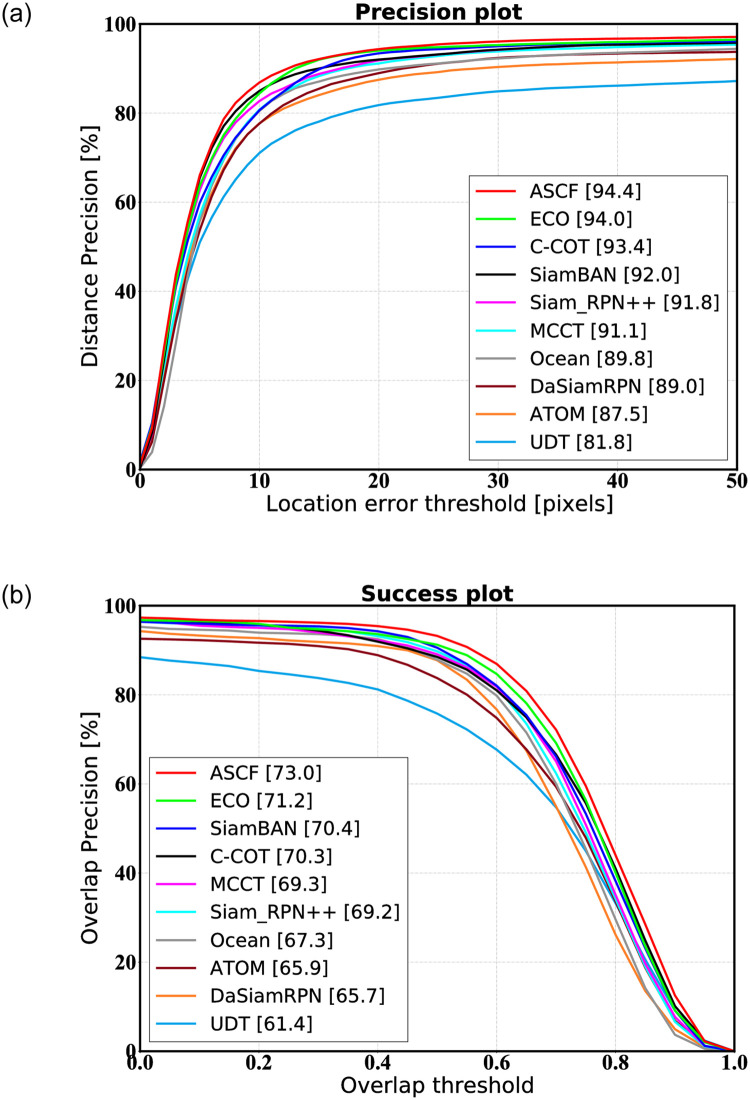
One-pass evaluation, the distance accuracy DP of the tracker and the area under the overlap rate curve accuracy AUC are displayed on the OTB-2013 data set, and the center position error CLF score threshold of the distance accuracy DP is set to 20. (a) OTB2013 (DP). (b) OTB2013 (AUC).

Fig 4 shows the DP and the AUC on the OTB-2013 dataset compared to the current state-of-the-art tracking algorithms, with the performance scores of each algorithm labeled in the legend. With an AUC of 73% and a DP of 94.4%, our algorithm is the best among the compared trackers in terms of both the AUC and DP evaluation metrics. Our tracker’s AUC is 0.4% higher than that of ECO when using the same correlation filter tracker, and its AUC is 2.5% higher overall. Compared with the Siamese tracker, our tracking algorithm improves more dramatically. Specifically, when compared to the Siamese tracker SiamBAN, which is the best-performing Siamese tracker on OTB 2013, the DP improved by 2.6%, and the AUC improved by 3.7%.

#### 4.1.2 OTB-2015

**Fig 5:**
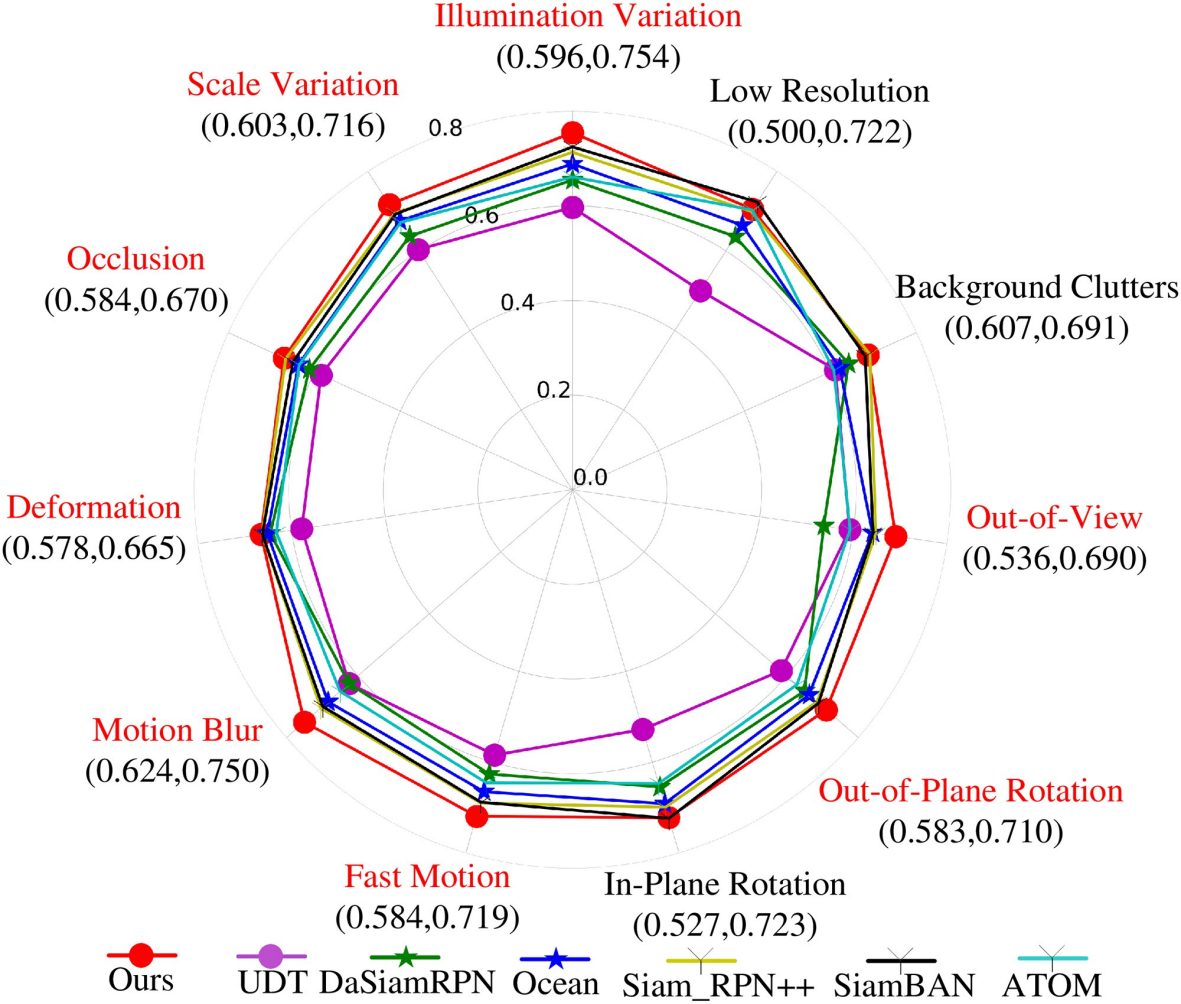
The AUC evaluation index values of each tracker under 11 different challenge factors in OTB-2015.

Compared with OTB2013, the OTB2015 [28] dataset is more difficult to track, and the tracking scenarios are more complex, which provides a relatively uniform testing and evaluation environment for tracking algorithms. We compare ASCF with the recent state-of-the-art trackers, including MCCT [14], C-COT [9], UDT [52], DaSiam [17], Ocean [40], Siam_RPN++ [54], ECO [10], SiamBAN [53], ATOM [16] and DiMP [18] on OTB2015. The comparison results are shown in Table 1. The ASCF tracker ranks first in terms of the AUC, DP and OP. Compared with the baseline tracker, ECO, ASCF improves the AUC, DP and OP by 3%, 0.3%, and 3.4%, respectively. Compared with the best performing Siamese tracker SiamRPN++, ASCF outperforms it in terms of the AUC, DP and OP by 2.4%, 0.3%, and 0.6%, respectively. The OTB2015 dataset divides the video sequence attributes in the test set into 11 categories according to common challenging factors in object tracking, including illumination variation (IV), deformation (DEF), scale variation (SV), occlusion (OCC), motion blur (MB), fast motion (FM), in-plane rotation (IPR), out-of-plane rotation (OPR), out-of-view (OV), background clutters (BC) and low resolution (LR), each video sequence in the test set contains at least one of the above properties. To further evaluate the effectiveness of our method in different tracking scenarios, we tested it in the above 11 tracking scenes. For the convenience of observation, six representative trackers were selected for comparison with the trackers in this paper. The evaluation results are shown in Fig 5. It can be clearly seen that the tracker proposed in this paper achieves a better AUC in most tracking scenes. Especially in terms of the MB, IV, OV, OPR, FM, DEF, OCC, and SV tracking scenes, it performs significantly better than the other algorithms. These experimental results show that our tracker can adapt to different common challenges in object tracking, and the adaptive update strategy is an important reason why the algorithm we propose can effectively address various challenges.

**Table 1:** Comparisons on OTB2015 dataset.

	UDT [52]	MCCT [14]	C-COT [9]	DaSiam [17]	Ocean [40]	ATOM [16]	Dimp [18]	ECO [10]	Siam_RPN++ [54]	SiamBAN [53]	ASCF
AUC	61.9	67.8	68.1	65.7	67.1	66.7	68.5	69.2	69.6	69.6	71.3
Precision	82.4	90.7	91.5	88.0	89.9	87.9	89.9	91.4	91.4	91.0	91.7
OP50	75.7	85.5	83.5	86.5	86.6	83.6	86.4	86.7	89.2	89.3	89.7

#### 4.1.3 GOT10K

GOT10K [27] is a large-scale dataset containing more than 10,000 videos that most deep learning trackers use for training. We evaluate the proposed algorithm in this paper on its test set, which contains a total of 180 videos with a total of 150 different categories, and we followed the evaluation guidelines and submitted the tracking results to GOT10k’s official online evaluation server. For the first time, this dataset combines categories with evaluation metrics, and it uses the mean average overlap (mAO) and mean success rate (mSR) as metrics. Compare with Ocean [40], DiMP [18], ATOM [16], TRASF [55], DPMT [56], SiamFC++ [39], SiamRPN++ [54], MemTracker [57], C-COT [9], ECO [10], SiamFC [34] and MDNet [58], which are the state-of-the-art trackers, the proposed algorithm performed well on the GOT10k dataset. Its average overlap (AO) and success rate (SR) are evaluated as shown in Table 2, and it can be clearly observed that the ASCF algorithm ranks first in terms of the mAO and mSR75 with values of 61.4% and 52.6%, respectively, which is better than that of DiMP, ATOM, and other deep correlation filter trackers.

**Table 2:** Experimental results on GOT10K dataset.

Tracker	Performance			Properties			Venue
	mAO	mSR50	mSR75	CF	Siamese	DL	
**ASCF**	0.614	0.696	0.526	✓		✓	
Ocean [40]	0.611	0.721	0.473		✓	✓	ECCV’2020
DiMP [18]	0.611	0.717	0.492	✓		✓	ICCV’2019
TRASF [55]	0.604	0.708	0.469			✓	ArXiv’2020
DPMT [56]	0.600	0.716	0.460	✓		✓	PRCV’2020
SiamFC++ [39]	0.595	0.695	0.479		✓	✓	AAAI’2020
ATOM [16]	0.556	0.634	0.402	✓		✓	CVPR’2019
SiamRPN++ [54]	0.517	0.616	0.325		✓	✓	CVPR’2019
MemTracker [57]	0.460	0.524	0.193			✓	ECCV’2018
C-COT [9]	0.406	0.415	0.161	✓		✓	ECCV’2016
ECO [10]	0.395	0.407	0.170	✓		✓	CVPR’2017
SiamFC [34]	0.392	0.426	0.135		✓	✓	ECCV’2016
ECOhc [10]	0.363	0.359	0.154	✓		✓	CVPR’2017
MDNet [58]	0.352	0.367	0.137			✓	CVPR’2016

The calculation formula of mAO is shown in the following Equation.

$$\begin{array}{c} mAO=\frac{1}{C}\sum_{c=1}^{C}(\frac{1}{\left|S_{c}\right|}\sum_{i\in S_{c}}AO_{i}) \end{array}$$ (19)

where *C* represents the number of types, *S*_*c*_ represents the number of video sequences under a certain type, and *AO* represents the average overlap. Similarly, the calculation formula of mSR is shown in Eq (20).

$$\begin{array}{c} mSR=\frac{1}{C}\sum_{c=1}^{c}(\frac{1}{\left|S_{c}\right|}\sum_{i\in S_{c}}SR_{i}) \end{array}$$ (20)

where SR represents the success rate.

#### 4.1.4 VOT2020

The visual object tracking challenge (VOT) is a recently released challenging target tracking evaluation dataset and is the most authoritative and influential evaluation platform dataset in the field of international object tracking. The difference from the previous VOT dataset is that the label format of VOT-ST2020 and VOT-RT2020 has changed from the original rotated rectangular box to a mask, where ST refers to short-term tracking challenges and RT refers to short-term real-time challenges. Inspired by AlphaRef, we introduced the AlphaRef mask branch to achieve mask segmentation of the tracking targets. We selected VOT2020-ST and VOT2020-RT to evaluate the tracker, and the evaluation indicators are the expected average overlap (EAO), accuracy, and robustness. Our tracker is compared with the following state-of-the-art trackers: AFAT [61], DPMT [56], DiMP [18], ATOM [16], CSR-DCF [59], SiamFC [34], TRASF [55], SiamMask [60].

**Fig 6:**
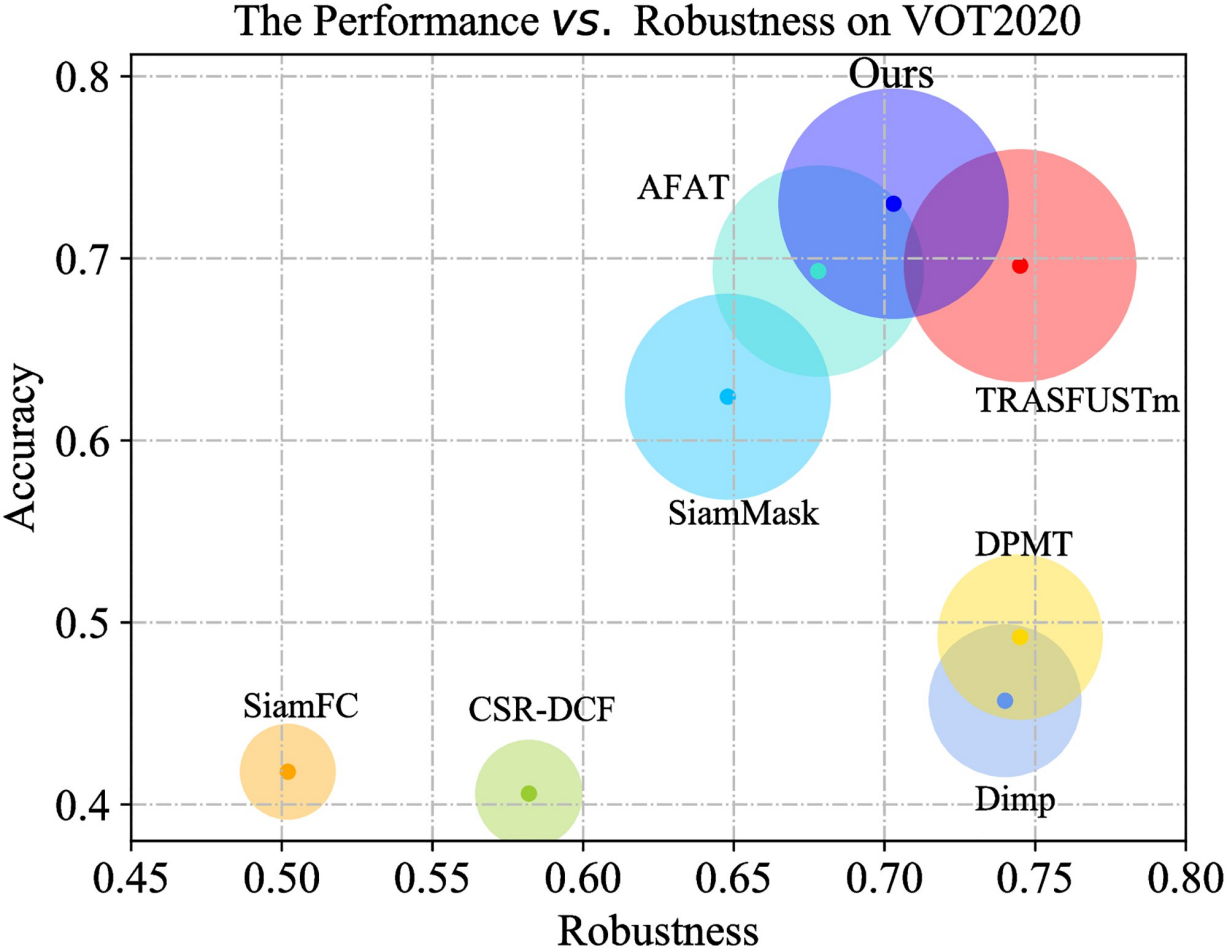
Comparison with state-of-the-arts on VOT-ST2020. We equip ASCF with a refinement module proposed by AlphaRef [62] to generate segmentation masks.

As shown in Fig 6, we visualized the accuracy and robustness of each tracker on the VOT-ST2020 short-term tracking challenge. Our tracker ranks first on the accuracy evaluation metric with a value of 0.73; the tracker proposed in this paper also achieved a robustness score of 0.703, which is higher than that of most of the compared trackers. This result verifies that our tracker can maintain good robustness while tracking with high accuracy.

As shown in Table 3, the EAO, accuracy, and robustness values of each tracker on the VOT-ST2020 dataset are shown. The results show that our tracker ranks second only to TRASF on EAO with a value of 0.396, which is 42.4% higher than the correlation filter tracker UPDT. In addition, the accuracy is 4.89% higher than that of the second-highest TRASF tracker among the compared trackers. At the same time, we made a comparison in VOT-RT2020, as shown in Table 4. There are trackers that perform well on short-term tracking challenges but not well in real-time challenges, such as the TRASF tracker. Our tracker achieves high scores on both short-term tracking challenges and real-time tracking challenges.

**Table 3:** Experimental results on VOT2020-ST dataset.

	SiamFC [34]	CSR-DCF [59]	ATOM [16]	Dimp [18]	UPDT [15]	DPMT [56]	SiamMask [60]	AFAT [61]	TRASF [55]	ASCF
EAO	0.179	0.193	0.271	0.274	0.278	0.303	0.321	0.378	0.424	0.396
Accuracy	0.418	0.406	0.462	0.457	0.465	0.492	0.624	0.693	0.696	0.730
Robustness	0.502	0.582	0.734	0.740	0.755	0.745	0.648	0.678	0.745	0.703

**Table 4:** Experimental results on VOT2020-RT dataset.

	SiamFC [34]	CSR-DCF [59]	ATOM [16]	UPDT [15]	Dimp [18]	TRASF [55]	DPMT [56]	SiamMask [60]	AFAT [61]	ASCF
EAO	0.172	0.193	0.237	0.237	0.241	0.282	0.293	0.320	0.372	0.332
Accuracy	0.422	0.405	0.440	0.443	0.434	0.576	0.487	0.624	0.687	0.639
Robustness	0.479	0.580	0.687	0.688	0.700	0.616	0.730	0.645	0.676	0.660

#### 4.1.5 LBT50

**Fig 7:**
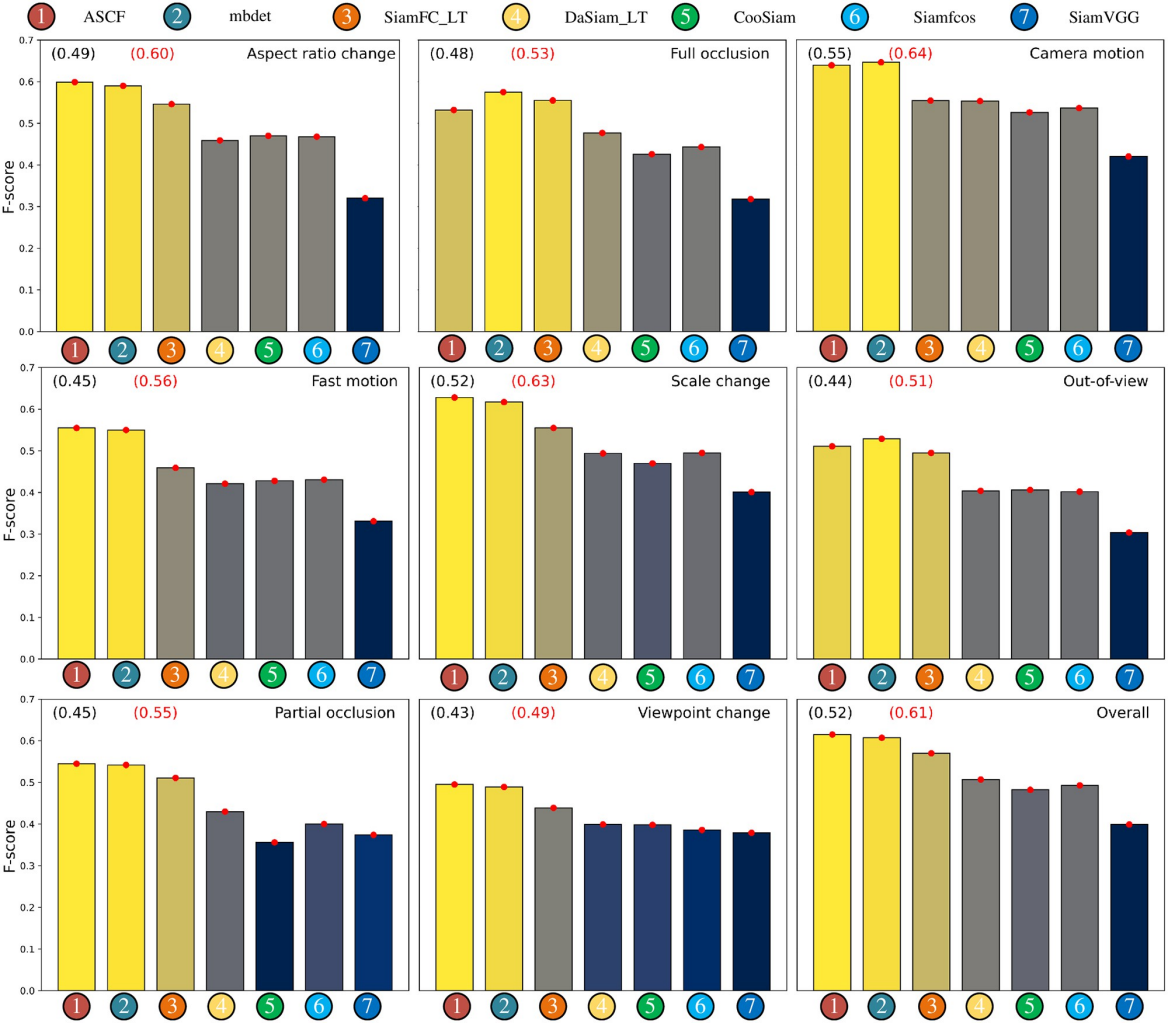
Attribute analysis on the LBT50 dataset.

LBT50 [30] proposed a long-term visual object tracking performance evaluation methodology and a benchmark and provides eight different long-term tracking challenge sequences. Fig 7 shows the F-scores over all 50 videos in the dataset. Our method achieves good results on all eight attributes but does not perform well on the out-of-view criterion because ASCF is not configured with a re-detection module. Our ASCF achieves an F-score of 61%, which is competitive with the other trackers. Our approach especially excels in the case of aspect ratio changes and scale variation, demonstrating the impact of our components.

### 4.2 Component-wise analysis

In this section, we choose to verify the effectiveness of the enhancements to the tracker performance of the proposed components in this paper on GOT-10K dataset.

#### 4.2.1 Deep feature

At present, most correlation filter trackers use the CNN to extract deep features and obtain tracking models through the learning and training of correlation filters. More specifically, we compare the baseline trackers with different deep features to verify the effectiveness of our used deep features. As shown in Table 5, Resnet50 [63], MobileNetv3 [64] and the Conformer network are used to extract the AUC and DP of the tracker (VGG16 was selected as the baseline). The experimental results show that the Conformer network can extract better-quality features in the image, so the AUC and DP of the tracker are improved.

**Table 5:** Analysis of deep feature on GOT-10K.

Comparison of features	mAO(%)	mSR50(%)	mSR75(%)
Baseline	39.5	40.7	17.0
Baseline+ResNet50	44.7	48.1	25.9
Baseline+MobileNetv3	38.2	37.3	16.4
Baseline+Conformer	47.3	54.6	28.5

#### 4.2.2 Model adaptive selection

As described in the section titled “Three-way parallel correlation filter tracking”, we trained a three-way correlation filter tracker and introduced spatial information. As shown in Table 6, the AUC of only using the Conformer network to extract features is 47.3%, and after adding the adaptive model selection strategy, the AUC improves significantly to 56.7%. This result verifies that the model adaptive selection strategy proposed in this paper can effectively improve the tracker’s accuracy.

**Table 6:** Component-wise analysis. Performance is evaluated on GOT-10K.

#Num	Metrics	mAO(%)	mSR50(%)	mSR75(%)
1.	Baseline	39.5	40.7	17.0
2.	+ Conformer	47.3	54.6	28.5
3.	+ Three-way track	56.7	63.2	46.8
4.	+ Adaptive update strategy	61.4	69.6	52.6

#### 4.2.3 Adaptive update strategy

As described in the section titled “Adaptive update strategy”, to selectively update the model in different situations, we propose a model adaptive update strategy. From the experimental results shown in Table 6, it can be seen that by using the combined Baseline + Conformer + Three-way track + Adaptive update strategy, the tracking accuracy and success rate of the tracker proposed in this paper are significantly better than the baseline or other combinations that use the baseline. Therefore, using an adaptive model update strategy can further improve the tracker’s performance.

### 4.3 Limitations of the proposed method

During the experiments, we found that the ASCF tracker still has some flaws. Although ASCF establishes tracking models in spatio-temporal locations and makes full use of space and time information, tracking drift still occurs when the model experiences tracking challenges such as long-term targets disappearing from the field of view for a long time. For example, ASCF does not perform well on the long-term tracking dataset LBT50 because ASCF lacks the re-detection mechanism implemented in the long-term trackers.

We tested the tracking speed of ASCF against the baseline tracker on the OTB2015 dataset, as shown in Table 7. The baseline and our proposed ASCF were both tested on the RTX 3070 GPU, and the tracking speeds with * are taken from the results published in the original paper. It can be seen that our tracker still has some defects in its tracking speed, so our next goal is to optimize the algorithm to improve the tracking speed.

**Table 7:** The amount of frames processed per second (fps) with different trackers.

Tracker	Baseline(ECO)	ASCF	DiMP	ATOM	Ocean
Tracking speed(Avg.FPS)	34	26	40*	46*	25*

### 4.4 Visualization

**Fig 8:**
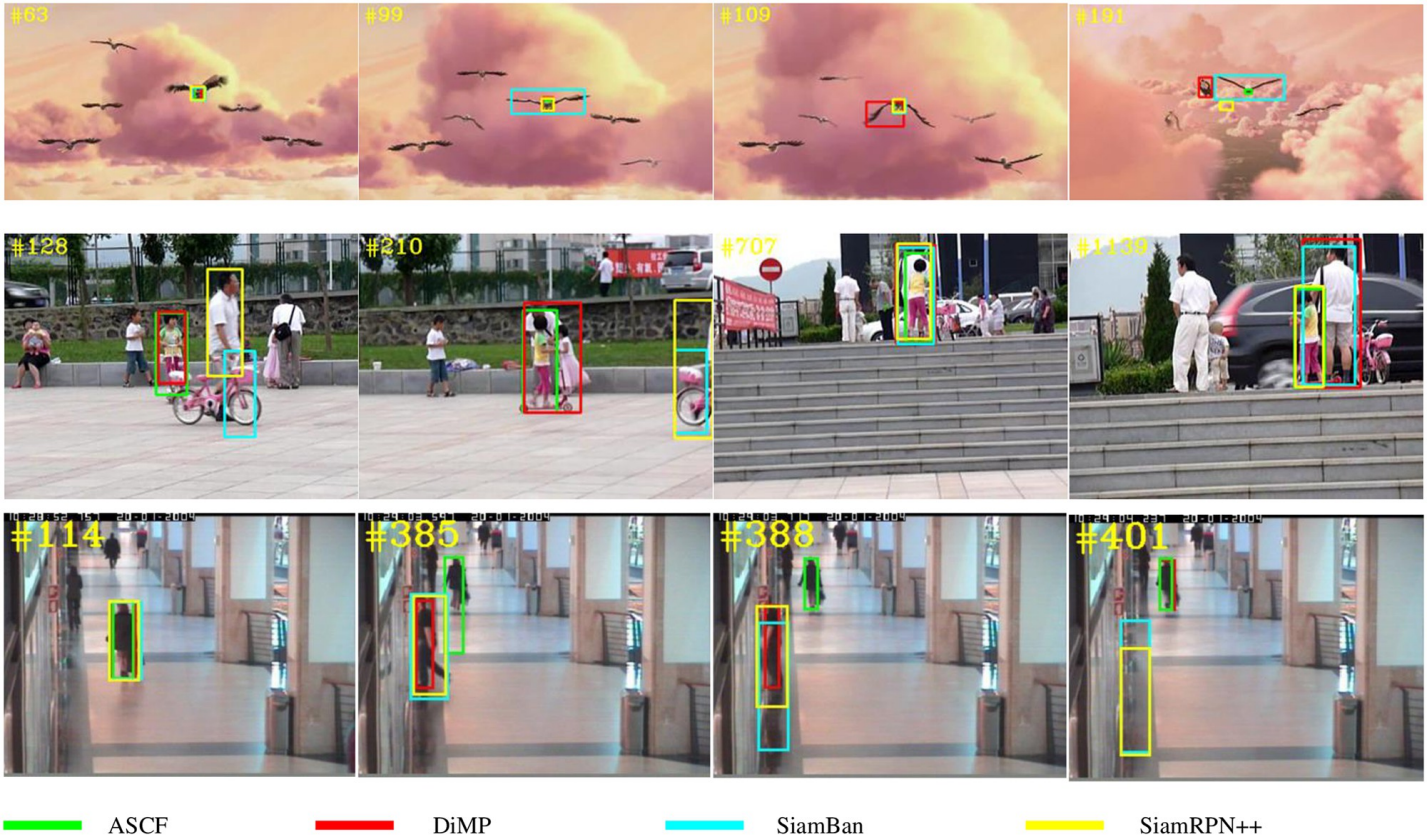
Tracking results on Bird1, Girl2 and Walking2 videos in OTB-2015.

We selected three representative trackers during the past two years as well as the tracking algorithm proposed in this paper for qualitative evaluation of some selected tracking sequences with different challenges. The results are shown in Fig 8. According to the visualization, our tracker is more robust compared to other trackers and produces more accurate tracking results when encountering occlusions, fast motion, and scale variation.

**Fig 9:**
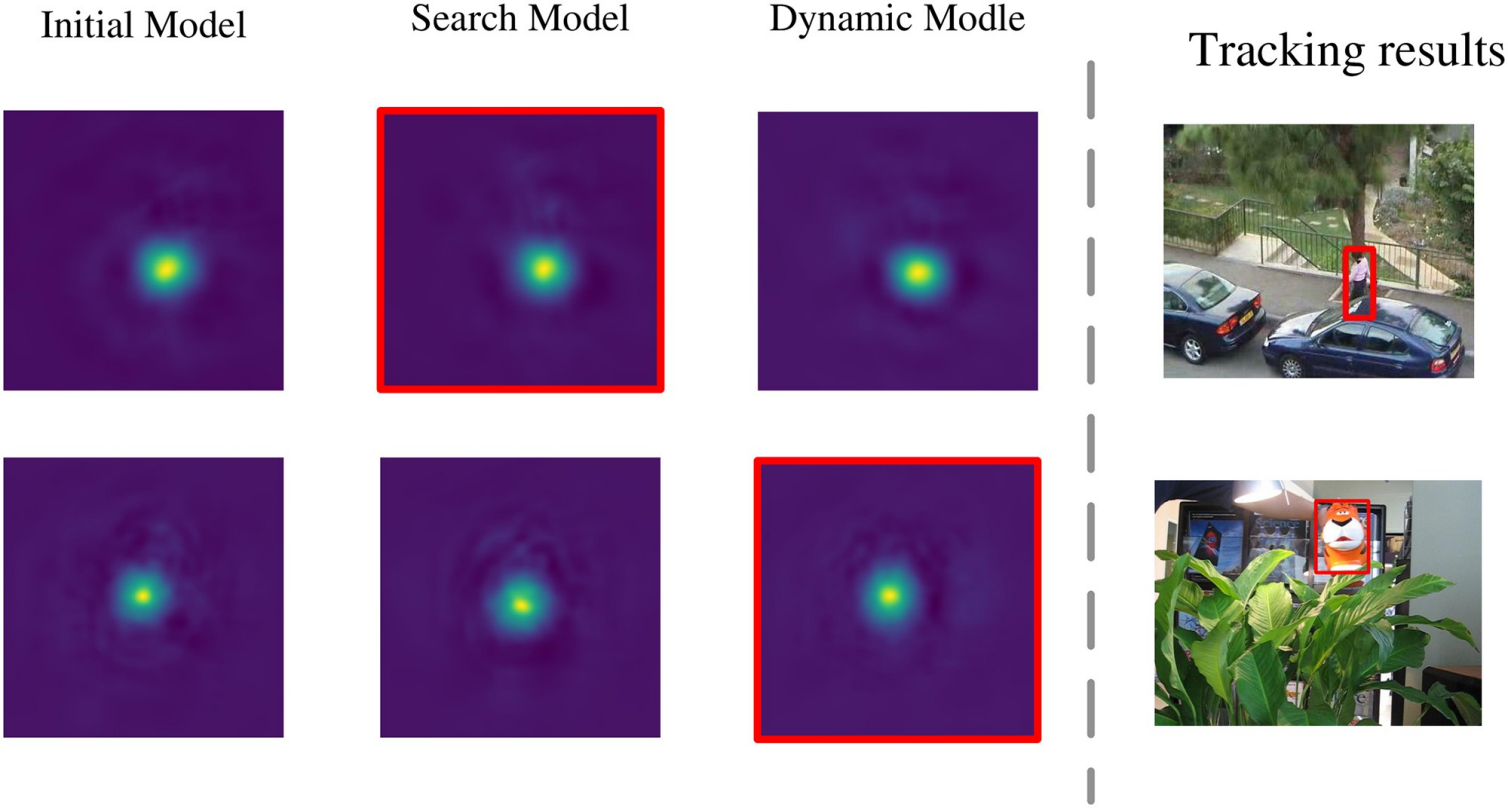
Visualization of response maps of different correlation filtering models. Selected models surrounded by red boxes. The results from top to down are Woman and Tgier1 from OTB-2015.

As shown in Fig 9, we made some visualizations for model selection and observed the tracking results generated by different correlation filtering models in the three channels. In the soccer tracking video sequence, due to the large deformation and occlusion of the tracking target and because the similarity to the initial image is low, it is necessary to update the correlation filter model with a faster frequency, and the current tracking frame uses a dynamic template model. In the basketball tracking video sequence, the tracking object in the current image frame has a high similarity with the initial tracking target, and the initial template correlation filtering model with higher confidence is selected for tracking.

## 5 Conclusions

In this paper, we propose a new ASCF tracking algorithm to spatio-temporally model the tracked target at different points in time and space during the tracking process. It consists of three correlation filters constructed with different spatio-temporal features, and the features are extracted by the Conformer network. The best tracking result is then selected by the adaptive model selection module proposed in this paper. Furthermore, we designed an adaptive model update strategy to avoid introducing disturbing information into the model. Finally, experiments were conducted on the public databases OTB2013, OTB2015, GOT-10K, VOT2020 and LBT50, and they demonstrate the superiority of ASCF and all of its components. By making full use of the spatio-temporal information through three different spatio-temporal tracking models, ASCF can track targets robustly and accurately in complex tracking environments. In future work, we plan to deploy our tracking framework on an end-to-end deep learning framework while improving the tracking efficiency to further improve the algorithm’s target tracking performance.
